# Link between concentrations of sediment flux and deep crustal processes beneath the European Alps

**DOI:** 10.1038/s41598-017-17182-8

**Published:** 2018-01-09

**Authors:** Philippos Garefalakis, Fritz Schlunegger

**Affiliations:** University of Bern, Institute of Geological Sciences, Baltzerstrasse 1+3, CH-3001 Bern, Switzerland

## Abstract

Large sediment fluxes from mountain belts have the potential to cause megafans to prograde into the neighbouring sedimentary basins. These mechanisms have been documented based from numerical modelling and stratigraphic records. However, little attention has been focused on inferring temporal changes in the concentrations of supplied sediment from coarse-grained deposits. Here, we extract changes of this variable in the field from a Late Oligocene, c. 4 km-thick suite of fluvial conglomerates situated in the North Alpine foreland basin, which evolved in response to the tectonic and erosional history of the Alps. We measure a decrease in channel depths from >2 m to <1 m and an increase in the largest grain sizes from <15 cm to >20 cm from the base to the top of the suite. These constraints are used to calculate an increase in fan surface slopes from <0.3° to >1.0° based on the Shields criteria for sediment entrainment. We combine slope and bulk grain size data with the Bagnold equation for sediment transport to infer higher concentrations of the supplied sediment. We use these shifts to propose a change towards faster erosion and a steeper landscape in the Alpine hinterland, driven by mantle-scale processes beneath the Alps.

## Introduction

Sediment flux has been considered as one of the major variables controlling the dispersion of sediment in sedimentary basins^1–3^ and has likewise assigned a primary role in regulating the fluvial style particularly in terrestrial environments^4^. In this context, it has been proposed that larger sediment fluxes tend rivers to adapt a braided pattern, while lower sediment discharge is commonly, but not always, associated with streams that are laterally confined by floodplains^4^. In the same sense, larger supply rates of sediment are capable of shifting sedimentary depocenters towards more distal sites, while reductions of sediment fluxes are commonly associated with a backstepping of sedimentary environments towards their sedimentary sources^1,5^. These mechanisms have been well documented from modern sedimentary systems and have particularly been inferred from stratigraphic records of coarse-grained clastic depocenters^3,6^, and they are decently well understood based on conceptual work^1,5^. However, sediment flux not only comprises a volumetric measure of the supplied grains, but it also includes the calibre and the concentration of this material^4^. While much attention has been paid on how shifts in grain size populations are recorded in sedimentary records, and on how these signals propagate through terrestrial environments^7–14^, much less research has been directed towards inferring possible changes in the concentrations of supplied sediment from stratigraphic records. However, because higher sediment concentrations in streams are consequences of faster erosion in the source area, as modern examples have shown^15^, this variable contains valuable information about the erosional processes that are dominant in the specific high relief erosional zone. Here, we extract sedimentary information from coarse-grained fluvial conglomerates that we use as a basis to infer changes in the concentrations of the supplied sediment. We focus on a suite of Late Oligocene conglomerates situated in the North Alpine foreland basin, which evolved in response to the tectonic and erosional history of the Alps. We use these shifts to propose a transient surface response of the Alpine landscape to fast rock uplift, driven by mantle-scale processes situated in the deep Earth beneath the Central Alps of Switzerland.

**Figure 1:**
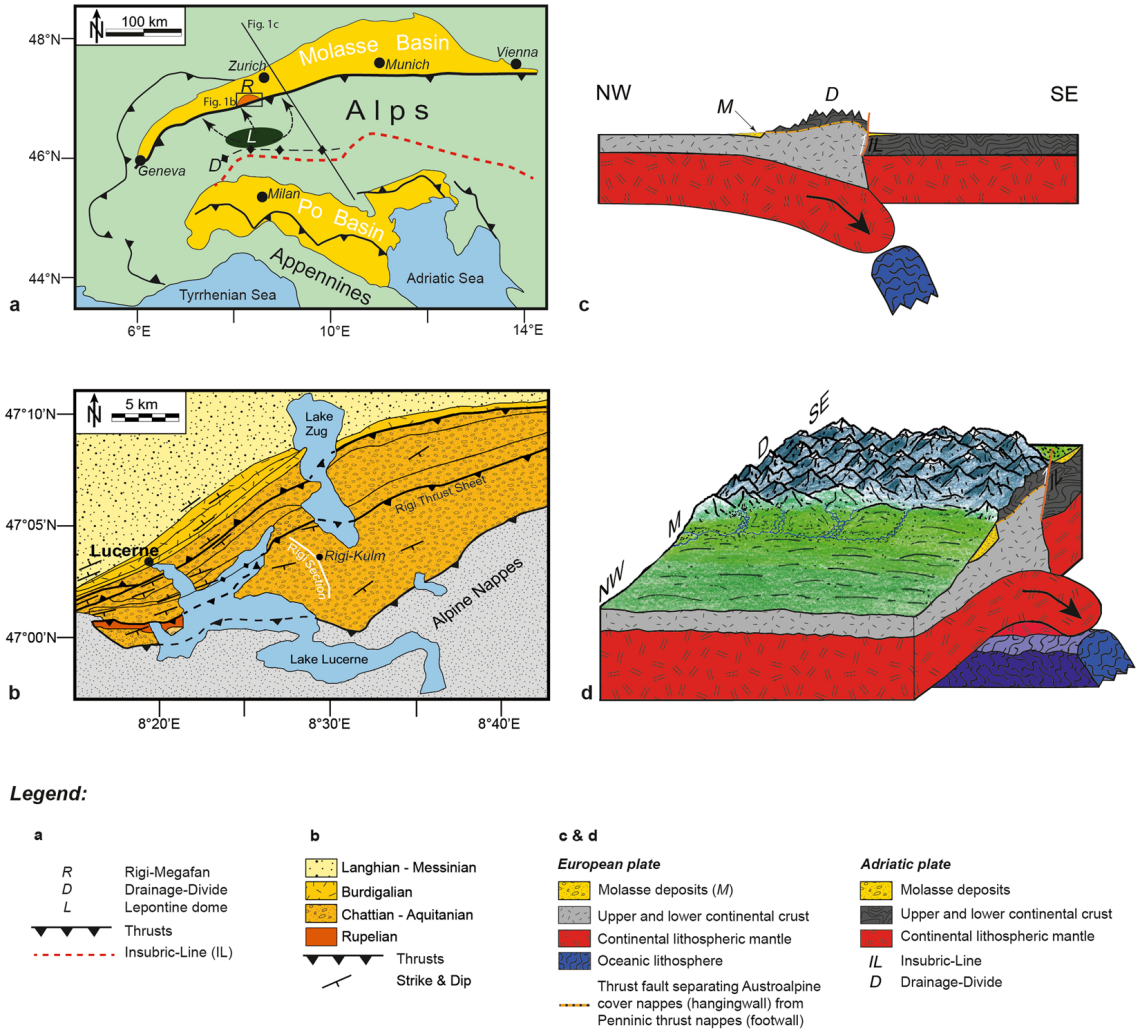
The Alps and their geodynamic state during the Late Oligocene. (**a**) Geologic map of the Alps and the neighbouring sedimentary basins^23^. The map also shows the location of the Rigi megafan (denoted as *R*) that was sourced from the area surrounding the Lepontine area (denoted as *L*) in the Central Alps of Switzerland. *D* denotes the major Alpine drainage divide between c. 30–20 Ma. (**b**) Simplified geologic map of the Rigi area, situated on the northern side of the Alps^22^. Please see Fig. 1a for location of map. (**c**) Schematic section through the Alps^23^ illustrating the geodynamic situation between c. 30–20 Ma. At that time, the Alps were made of the orogenic lid constituted by the Austroalpine nappes, and the crystalline core that assembled lithologies of the Penninic nappes. Slab breakoff beneath the rear of the Alps resulted in a period of fast rock uplift and in the formation of a highly elevated topography. Please see Fig. 1a for location of section. (**d**) 3D perspective on the Alps and the Molasse foreland basin illustrating the geodynamic situation between c. 30–20 Ma. Streams with sources in the Central Alps deposited large megafans at the thrust front. These were laterally bordered by bajada fans with sediment sources situated in the frontal Alpine nappes. The discharge within the Molasse foreland basin was oriented towards the NE at that time. Figures a) through c) have been drawn based on Schlunegger and Castelltort^23^ using Illustrator 15.1.0 licensed to Uni Bern.

The Swiss Alps (Fig. 1a) can be characterized as a doubly-vergent mountain belt with a crystalline core exposed in back of the orogen that is flanked by N- and S-vergent thick-skinned and thin-skinned thrusts^16^. The present-day litho-tectonic architecture of the Alps resulted from the subduction-collision history between Mesozoic and Neogene times^16^. It started with the subduction of the European oceanic lithosphere beneath the Adriatic continental plate and resulted in the closure of the Tethys Ocean sometime during the Late Cretaceous^16^. At c. 35 Ma, the collision between both continental plates was completed and followed by the delamination of the European oceanic lithosphere slab beneath the core of the Alps^16,17^. The result was rapid rock uplift and orogen-parallel extension in the rear of the Alps^16^. This was also associated with the build up of topography to the current elevations^18^, which in turn caused an increase in the erosion rate and the sediment flux to the adjacent sedimentary basins^19^.

The Molasse foreland basin, situated on the northern side of the Alps (Fig. 1a,b), records the erosional evolution of this orogen^16^. Surface uplift in the back of the Alps following slab breakoff (Fig. 1c) and the related increase in sediment discharge to the basin^19^ was considered to have caused the change from the early underfilled ‘flysch’ to the subsequent overfilled ‘molasse’ type of sedimentation^20^. This stratigraphic change occurred at c. 30 Ma, as revealed by magneto-polarity stratigraphies^21^. During Molasse times when overfilled conditions prevailed, large sediment fluxes resulted in the construction of alluvial megafans^3^, which expanded radially into the foreland basin over several tens of kilometers. Close to the apex at the proximal basin border, the megafans are laterally embedded by locally-derived ‘bajada’ fans^21^. While these bajada systems were mainly sourced in the frontal Alpine nappes, the material of the megafans was derived from both the northern Alpine border and the core of the Alps surrounding the Lepontine dome (Fig. 1a)^21,22^.

Here, we focus our analyses on the Late Oligocene Rigi megfan conglomerates situated in the north Alpine foreland basin at 47°03'N / 8°29' E (Fig. 1a,b). These deposits chronicle the rise of the Alpine topography and the related changes in erosional mechanisms between 30 and 25 Ma^23^. We present data on the structure (e.g., cross-beds, basal contacts, palaeochannel depths) and the grain size of these sediments in order to calculate the fan morphometry including the fan surface slope. We then combine these data with published information about sediment accumulation rates to infer changes in both the rates and the concentrations of the supplied sediment through time. We finally complemented the stratigraphic and sedimentologic dataset with published information about shifts in petrofacies to infer a transient surface response to slab breakoff in the back of the Alps (Fig. 1c,d). We reveal that data about the grain size and the sedimentary structures of conglomerates within a detailed chronological framework^22^ are essential for linking processes on the Earth’s surface with the subduction mechanisms in the mantle.

## Results

**Figure 2:**
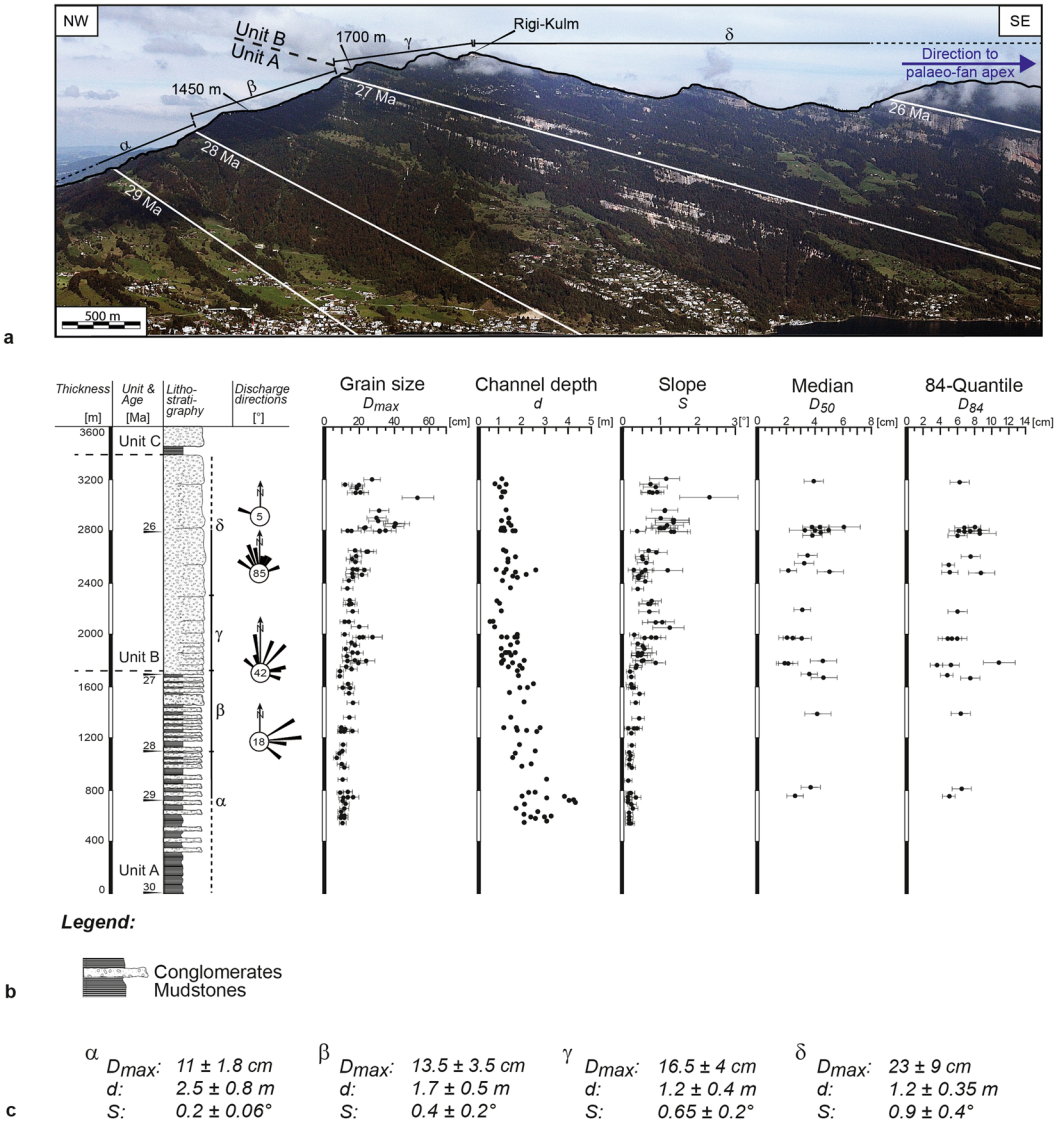
Stratigraphic architecture of the Rigi megafan. (**a**) Photo showing Rigi Mountain. The fan apex deposits crop out on the SE margin of the photo. Accordingly, the deposits at the top of the section (part δ) chronicle a successively more proximal situation. This could potentially blur the interpretation of a progradational trend recorded by the stratigraphic trend of the section. Nevertheless, because shifts towards a more proximal sedimentary environment occurs also on the steep ridge, displayed on the NW margin of the photo (between parts α and γ), the coarsening- and thickening-upward record does include a progradational trend of the Rigi megafan. (**b**) Stratigraphic section^22^ that is based on a compilation from field-based investigations^24^. The ages are based on magneto-polarity stratigraphies and have been used to correlate the Rigi conglomerates with other sections in the foreland basin. The figure also shows the results of the sedimentologic investigations, including trends in grain size, channel depths, and palaeo-slopes. Palaeoflow data is based on orientations of gutter casts and cross-beds that we have measured in the field. The number in the circle of the rose diagrams indicates the number of observations. These measures have been combined with literature data^22,24^ into representative rose diagrams. We note that post-depositional compaction could bias the measured channel depths. However, related effects are expected to be larger where the channels are eroded into mudstone (unit A), because mudstones are likely to be compacted to a higher degree than the conglomerates. Therefore, it is likely that underestimates of channel depths in unit A would be larger than in unit B, which contains conglomerates only. The effect is an amplification of the decreasing channel-depth trend illustrated in Fig. 2b. (**c**) Summary of results (largest grain size *D*_*max*_, channel depth *d*, slope *S*) for the base (α) and the top (β) of unit A, and also for the base (γ) and the top (δ) of unit B. The subdivisions into α, β, γ and δ of the Rigi section are also displayed on the photo (Fig. 2a) and for the section (Fig. 2b).

Among the various sites where alluvial megafan conglomerates have been mapped in the Swiss Molasse, the section at Rigi (Fig. 2a) offers the best exposure where a 30 to 25 Ma-old continuous suite of proximal megafan deposits are encountered. There, the deposits can be grouped into three units, which we refer to as units A to C (Fig. 2b) based on characteristic alternations of lithofacies and the occurrence of key clast types in the conglomerates. Unit A comprises the lowermost 1700 m of the Rigi section and displays an alternation of several m-thick conglomerate beds and mudstone interbeds^24^. Clast types mainly include carbonate constituents including dolomites, micritic and siliceous limestones, and sandstones that were derived from the frontal Alpine nappes at that time^24^. Magneto-polarity chronologies reveal that unit A chronicles the evolution of the megafan between 30 and 27 Ma^22^, and that sediment accumulated at rates between 500 and 600 m/My^22^. The second unit B, encompassing the thickness interval between 1700 m and 3400 m of the section, comprises an amalgamated stack of dm- to m-thick conglomerate beds. It is characterized by the occurrence of granite clasts that were most likely derived from the crystalline core situated in the rear of the Alps during the Late Oligocene^24^. Magneto-polarity chronologies reveal that this unit was deposited between 27 and 25–26 Ma^22^. However, age assignments for the top of unit B strongly depend on how the Rigi magneto-polarity stratigraphy is correlated with the magneto-polarity time scale^22^. Accordingly, rates of sediment accumulation vary from 800 m/My to >1500 m/My during the time span of unit B deposition^22^. The deposits of the uppermost c. 200 m of the section, here referred to as unit C, are characterized by alternating mudstones and 3–5 m-thick conglomerate beds with a monomict composition where Penninic flysch clasts are the dominant constituents^24^. Because Penninic flysch nappes most likely formed the Alpine border during Late Oligocene times, as palinspastic restorations revealed^25^, unit C had a local provenance^22,24^. The next paragraphs present the sedimentologic details of each unit.

### Base of the section – unit A

Unit A is made up of an alternation of conglomerate beds and mudstone interbeds (Fig. 2b). Individual conglomerates are up to 15 m thick and comprise amalgamated stacks of 2 to 3 m-thick beds that are separated by erosive contacts with m-deep scours. The conglomerates are clast-supported and comprise a massive fabric in most places. Locally, cross-beds with several meters-wide diameters are also visible. Channel depths *d* scatter around 2–3 m. Individual shallow and deep scours (<1.5 m and >4 m, respectively) also occur. The sizes of the largest clasts *D*_*max*_ range between 10–15 cm, while the *D*_50_ and *D*_84_ are between 2–6 cm and 3–8 cm large, respectively. The shape of individual clasts range between subrounded and rounded if the classification scheme of Tucker^26^ (1988) is used for comparison purposes. Mudstone interbeds comprise dm- to m-thick sequences of parallel laminated and cm-thick units. Ripple marks, bioturbation, slickenside fabrics, rootlets and yellow-brown mottling together with caliche nodules also occur. Occasionally, cm-thick mudstones display a dark brown to black colour and contain bone fragments of micro mammals in a few places^22^.

Petrographic investigations^24^ revealed that unit A clasts comprise limestone constituents at the base and radiolarite cherts towards the top of the section. Both clast types were most likely derived from the Penninic (limestone clasts) and Austroalpine cover nappes (limestone and radiolarite clasts) that were exposed at the orgenic front prior to 25 Ma according to palinspastic restorations^16^. Orientations of gutter casts and axes of cross-beds (Fig. 2b) imply a palaeo-discharge direction that was oriented towards the ENE, i.e., parallel to the basin’s axis.

Calculations of fan surface slopes returned values that are generally low. In particular, most of the values range between 0.2° (part α, base of unit A) and 0.4° (part β, top of unit A; Fig. 2b,c). Note, the variations in channel depths *d* and the spread and uncertainties in the *D*_*max*_ values have a minor effect on the results of the fan surface slope calculations. The largest contributions to the scatter in the slope data are due to the two fold differences in assigned values for the Shields^27^ variable *ϕ*.

### Middle part of the section – unit B

The unit B suite comprises an amalgamated stack of approximately 80 cm- to c. 1.5 m-thick conglomerate beds, forming up to 30 m-thick conglomerate packages (Fig. 2b). Interbedded mudstone beds are rare, and if present they are <1 m thin. The conglomerates reveal a clast-supported and massive fabric with imbrications in places. Where visible, cross-beds with several meters-wide diameters and up to 1.5 m heights also occur. Individual channels were generally 1 m deep. In some places, erosional scours are shallow and only 50 cm deep. At sites where conglomerates are arranged as cross-beds, channel depths exceed 2 m. This has particularly been observed in the lower part of unit B. The sizes of the largest particles *D*_*max*_ range between 20–40 cm, while the diameters of the *D*_50_ and *D*_84_ measure between 2–6 cm and 3–11 cm, respectively. The clasts are generally subangular if the classification scheme of Tucker^26^ is used as a basis. Mudstones interbeds comprise cm- to dm-thick sequences of red and yellow mottled units preserving parallel laminations and ripple cross-beds in a few places only. Most of the mudstone interbeds, however, have a massive fabric with slickenside fabrics and caliche nodules.

Petrographic investigations^24^ revealed that unit B conglomerates contain red granite clasts that were derived from the basement of the Austroalpine nappes forming the orogenic lid during that time, as revealed from palinspastic restorations of the Central Alps^16^. Orientations of imbrications and axes of cross-beds reveal a palaeo-discharge direction that was oriented towards the NNW, i.e., perpendicular to the basin’s axis.

During accumulation of unit B, however, fan surface slopes increased from a mean value of c. 0.65° at the base of unit B (part γ of the Rigi section) to >1° towards the top (part δ), albeit with a large scatter (Fig. 2b,c). The steepest slope >2°, calculated for a channel close to the top of unit B, could be related to the foothill of the bordering bajada fans (see below) where distal debris flow processes might have reached the surface of the Rigi megafan.

### Top section – unit C

Unit C is characterized by an alternation of mudstones and 3–5 m-thick conglomerate beds with a monomict composition where Penninic flysch clasts are the dominant constituents^24^. The clasts are generally angular to subrounded. The ensemble of these observations has been used to assign this unit to a bajada-type environment^22^. Sediment sources were situated at the Alpine front, from where the material was supplied through debris flows processes and high-concentrated floods^21^. Because of the local character of these deposits and since mass wasting processes cannot be described with the Meyer-Peter and Müller^28^ principles for sediment transport, we will not consider unit C conglomerates in the further analysis.

### Summary: evolution from the base to top of the Rigi megafan

In summary, the change from conglomerate-mudstone alternations at the base to the construction of amalgamated conglomerates towards the top of the section at Rigi was associated with a decrease in channel depths from >2 m to <1 m, albeit with a large scatter. These shifts in the stratigraphic architecture were associated with changes in palaeo-flow directions from an axial- to a radial-directed dispersion pattern (Fig. 2). The largest changes in grain size are observed for the *D*_*max*_, which increases from c. 10–15 cm at the base of the sequence, to approximately 20–40 cm towards the top. The mean grain size *D*_50_ remains stationary between 2–6 cm, while the *D*_84_ increases slightly from 3–8 cm (with a mean of c. 5.5 cm) at the base to 3–11 cm (with a mean of c. 7 cm) towards the top of the Rigi megafan conglomerates. In addition, the roundness of clasts shifts from between subrounded and rounded at the base of the conglomerate suite to subangular towards the top.

Calculations of fan surface slopes (eq. 6) reveal a large spread, which we partly explain by the scatter in the *D*_*max*_ datasets and the measured channel depths *d*. The largest uncertainties, however, result from assignments of values for the Shields^27^ variable *ϕ*. They can vary by up to a factor of 2 depending on reach specific arrangements of clasts (loose and locked boundaries between clasts including hiding and protrusion effects, see methods). While we cannot fully address these complexities with field-based observations, we note that equation 6 returns a trend towards steeper fan surfaces from the base to the top of the section. In particular, the shifts in sedimentation patterns were associated with a >200% steepening of the fan surface from originally <0.3° at the base to >1° towards the top of the conglomerate suite (Fig. 2).

## Discussion

The coarsening- and thickening-upward megasequence suggests that the depositional style changed through time from a distal position to a more proximal environment on the megafan^3,4,21,29^. Supporting evidence is provided by the change in the stratal architecture, the sedimentary fabric and the trend toward less rounded clasts from the base to the top of the Rigi conglomerates. This change in sedimentary fabric and depositional processes was also associated with (i) an upsection trend towards a steeper slope of the fan surface, (ii) an increase of the largest clasts, and (iii) a decrease in channel depths. Such a change in the stratigraphic record could either be explained by the exposure situation where c. 26.5 Ma-old conglomerates (at Rigi-Kulm, Fig. 2a) are farther away from the palaeo-fan apex than the 26 Ma-old deposits^24^ (on the SE corner of Fig. 2a), or by megafan progradation^1,5^. However, shifts towards a more proximal environment, where conglomerate beds are more frequent in the stratigraphic suite, are also observed along the steep ridge to the NW of Rigi-Kulm, exposing sediments of parts α through γ in their vertical position (see Fig. 2a). In addition, coarsening- and thickening-upward megasequences are also recorded at contemporaneous sections >15 km farther to the northeast that chronicle the sedimentation pattern of the same dispersal system^22^, but in a more distal position^22^. Accordingly, while we cannot fully exclude the possibility that the exposure condition partially explains the aforementioned stratigraphic architecture, the coarsening- and thickening-upward record does include a progradational trend of the Rigi megafan.

Megafan progradation can be explained through: (i) the northward propagation of the Alpine mountain belt and thus of the orogen front^16,22,25,30^, (ii) a decrease in sediment accumulation rates at constant sediment supply rates^5^, or (iii) an increase in sediment flux at constant or increasing sediment accumulation rates^1,5,7^. We acknowledge that we cannot fully exclude option one as a driving force for the progradational trend of the megafan, because the Alpine front did shift northward during that time^25^. However, we do not consider that this mechanism alone explains the inferred shifts towards more proximal sedimentation, because the progradation velocities of the Molasse gravel fronts were faster than the progradation rate of the Alpine thrust front, as palinspastic restorations revealed^20^. In the same sense, we reject option two as a conceivable mechanism because sediment accumulation rates were increasing and not decreasing up-section, or at least remained constant^22^. Rather, we consider the change towards more proximal sedimentation paired with the augmentation of the depocenter surface slope, and the increase in maximum grain size as a consequence of a higher sediment supply rate *Q*_*s*_^4^. This interpretation is consistent with the results of numerical models where large-scale coarsening- and thickening-upward megasequences at constant, or increasing sediment accumulation rates point towards a flux- rather than a tectonic-driven change^1,5^. It appears that such a flux-driven surface response was large enough to result in the burial of the forebulge in the distal foreland, as palaegeographic reconstructions have shown^31^. This allowed the sediment to disperse over a broader area and to change the sediment dispersion from an axial to a radial pattern^3^. We thus use the ensemble of these observations to propose that megafan progradation at Rigi was to a large extent driven by an increase in sediment discharge and not primarily by tectonic processes, as previous authors have claimed^21,22,24,25^.

Here, we provide evidence for proposing that the temporal increase in sediment flux was also associated with a higher concentration *Q*_*c*_ of the supplied sediment. In this context, we recall that at transport-limited conditions, which is usually the case where megafan progradation is flux driven^1,3,4^, volumes of supplied sediment per unit time depend on stream power^5,32^. This variable can be expressed, in the simplest case, as the product between water discharge *Q* and energy gradient or surface slope *S*. These relationships, which have been proposed as early as 1955 by Lane^33^, have also been derived on theoretical grounds^5,34^. In addition, they are based on the Bagnold^35^ bed-load transport formula, and the Darcy-Weisbach equation for computing bedform friction effects on stream flow. Finally, correlations between channel widths and magnitudes of channel forming floods^36,37^, which commonly correspond to water fluxes during bankfull discharge *Q*_*b*_ events, have also been considered to derive the dependency of sediment flux on stream power^5,32^. Furthermore, *Q*_*b*_ floods are likely to be strong enough to accomplish equal mobility conditions for all grain sizes, as modern examples of streams in the Swiss Alps have shown^38^. Accordingly, the relationship between stream power and sediment transport then takes the form:

1 $${Q}_{b}S=k\,\ast \,{Q}_{s}\sum _{i}{p}_{i}{D}_{i}$$

where *k* denotes a proportionality constant, *D*_*i*_ represents the grain size of a particular percentile, and *p*_*i*_ the probability of its occurrence within a grain size population. In addition, it was proposed that at equal mobility conditions, the bed sediment mixture can be reasonably well represented by a single grain size only^39^. Among the various percentiles representing the grain size *D* at equal mobility conditions, the *D*_50_ has been considered to best represent the complete range of grain sizes^39^. Alternatively, the *D*_*84*_ could be of particular interest because this percentile characterizes the bed roughness and the gravel bar structure^40–42^. Accordingly, eq. (1) simplifies to the following expression:

2 $${Q}_{b}S=k\,\ast \,{Q}_{s}{D}_{50}$$

Also at transport limited conditions, an increase in stream power results in a larger flux and calibre of the supplied sediment, where:

3 $${\rm{\Delta }}({Q}_{b}S)=k\,\ast \,{\rm{\Delta }}({Q}_{s}{D}_{50})$$

Regrouping of eq. (3) and expressing sediment concentration *Q*_*c*_ as the ratio between sediment and water flux *Q*_*c*_ = *Q*_*s*_ */Q*_*b*_ finally returns an expression that approximates, at first order, the temporal change in *Q*_*c*_, where:

4 $${\rm{\Delta }}{Q}_{c}=k{\rm{\Delta }}\frac{S}{{D}_{50}},$$

or alternatively

5 $${\rm{\Delta }}{Q}_{c}=k{\rm{\Delta }}\frac{S}{{D}_{84}}$$

Here, *k* drops out because it is a constant^5^. Equations (4) and (5) then predict that a change in the concentration of the supplied sediment is proportional to a shift in the ratio between the fan surface slope and the calibre of the supplied sediment. At Rigi, the up-section increase of both grain size percentiles (~20%, from c. 5.5 cm to 7 cm for the *D*_*84*_ and zero for the *D*_*50*_) is much less than the change in fan surface slope *S*, which steepened by >200% form <0.3° to >1° (Fig. 2). The consequence is a substantial increase in sediment concentration *Q*_*c*_ through time (eqs 4 and 5).

Here, we use the inferred increase in the flux *Q*_*s*_ and the concentration *Q*_*c*_ of the supplied sediment as a basis to propose a change towards faster erosion, and related to this, towards a possible change of the landscape shape in the Alpine hinterland. In this context, we recall that in general, sediment and water flux both increase with the size of the drainage basin *A*. In addition, both values can be computed, in the simplest case, through the multiplication of *A* with erosion rate *E* and precipitation rate *P*, respectively^34,43^. Accordingly, an increase in the concentrations of the supplied sediment could then be explained by a decrease in precipitation rates, while erosion rates remained constant. However, such a scenario is very unlikely because neither palaeo-floral^44^ and isotope records from Molasse sediments^44^, nor palaeo-ecological proxy data from deposits in northern continental Europe^45^ do provide evidence for a change in local and regional palaeo-climates, including precipitation^44,45^ during the time when the Rigi conglomerates accumulated^23^. Furthermore, shifts towards a warmer palaeoclimate occurred at 25.5 Ma^23^, thus 1.5 My later than the change from unit A to B. We rather suggest that the shifts towards larger sediment concentrations, paired with larger sediment fluxes, point towards an increase in erosion rates during the Late Oligocene. We also propose that this inferred increase in erosion rates was associated with a steepening of the Alpine landscape in response to rock uplift driven by lithosphere-scale processes, as outlined in the next paragraph.

**Figure 3:**
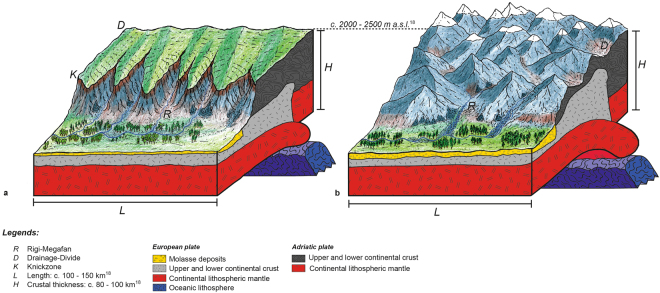
Topographic development of the Alps in response to slab breakoff. (**a**) Situation following slab breakoff. Incipient rise of the Alpine topography caused fluvial incision to initiate at the orogen front as revealed by petrographic investigations and palinspastic reconstructions^23–25^. This most likely reflects the occurrence of a dissected transient landscape at the orogen front, while the headwaters represented a flat-laying, non- to poorly dissected plateau as clast types from farther south are absent in pre 27 Ma-old deposits^24^. We suggest that the flat headwater reaches in the south, and the dissected Alpine landscape farther north were separated by a knickzone (*K*) where most of the erosional work was accomplished. We use this scenario to explain the relatively low fluxes and concentrations of supplied sediment. In the Molasse foreland basin, low sediment fluxes resulted in the establishment of a relatively flat fluvial environment with channels that were confined by a floodplain. See text for further explanations. (**b**) Ongoing uplift promoted headward retreat of knickzones and the shift of the erosional front towards the drainage divide (D) situated in the rear of the Alps, thereby reaching the region where crystalline rocks were exposed. At this stage, the Alpine landscape has reached a steady state situation where ongoing rock uplift is fully compensated by erosion^18^, and where the shape of the Alpine landscape has remained stationary. This mechanism explains the delayed arrival of crystalline clasts in the conglomerates and the increase in both the flux and the concentrations of supplied sediment. Sediment concentrations increased until this steady state situation was established. In the Molasse foreland basin, the ensemble of these processes finally caused the Rigi megafan to prograde. See text for further explanations. Please note that both figures have the same scale. Please also note that the topography is exaggerated for illustration purposes. The view on both illustrations is towards the SE.

The geodynamic evolution of the Alpine orogen can be characterized through the subduction of the European plate beneath the Adritatic continental microplate, which started in Late Cretaceous times^16^. Ongoing subduction and closure of the Tethys finally resulted in the collision between both continental plates^16^. This was accomplished at c. 35 Ma, when the buoyant European continental plate entered the subduction channel. The differences between the buoyancy forces of the continental part of the European plate and the vertically-directed slab load forces of the subducted oceanic European plate resulted in differential forces, with the result that the subducted oceanic slab broke off between 32–30 Ma^16,17^ (Fig. 1c,d). Slab breakoff was followed by rapid rock uplift near the southern limits of the Central Alps and the rise of the Alpine topography to their current elevations between c. 30–25 Ma^18^, exactly at the time when the Rigi conglomerates were deposited. Accordingly, we infer that slab breakoff and related surface uplift was the principal driving mechanism for the depocenter progradation at Rigi. We see this mechanism as delayed, and thus transient, surface response where the Alpine streams reacted to the rise of the Alpine topography through headward retreat (Figs 3a,b). The related surface response to slab delamination processes are outlined in the next section.

Channels respond to an increase in rock uplift through steepening their gradients and through an amplification of erosion^46^. If these changes occur in pace with the acceleration in rock uplift rates, i.e., at steady state, then sediment discharge and sediment concentrations will increase at the same pace. Although we cannot discard this possibility with the available grain size dataset, we prefer a transient response to rock uplift. In particular, the predominant occurrence of sedimentary clast types at the bottom of the Rigi section^24^ suggests that erosion initially started at the orogen front where these lithologies were exposed at that time^25^. This resulted in the formation of a landscape, where the northern margin of the Alps was characterized by the occurrence of deeply dissected valleys. Farther to the south, the Alpine landscape at that time can be described as a flat-laying, much less dissected plateau^18^ (Fig. 3a), similar to the non-dissected, low relief landscape fragments in the modern Central Andes^47,48^. At 27 Ma, at the base of unit B, the arrival of crystalline clasts in the conglomerates^24^ suggests that erosion started to affect the Alpine landscape near the drainage divide situated in the rear of the Alps (Fig. 3b), where the corresponding crystalline rocks were exposed at that time^16^. Such a southward shift of the sediment sources thus reflects a headward retreat of an erosional front, which was possibly delineated by a knickzone (Fig. 3a). This transient surface response to rock uplift was also associated by an increase in both the flux and the concentration of the supplied sediment (see above), which ultimately reflects a change in the erosional pattern in the Alpine hinterland and a shift towards a steeper landscape (Fig. 3).

If our interpretation is correct, such a proximal record of transient erosional response offers a laboratory for exploring sediment-flux and sediment-concentration dependent surface processes both in the erosional hinterland and in the foreland. Essential in this context are two sets of non-related pieces of information, which includes a temporal calibration of the deposits through detailed chronologies, and a quantification of surface slopes. Indeed, changes in stacking patterns of conglomerates within a detailed chronological framework yields in the identification of shifts in the flux of the supplied sediment only, but this variable alone has no predictive power on the concentrations of the supplied sediment. Contrariwise, modifications in fan surface slopes provide the basis to estimate shifts in stream power recorded by stratigraphy. The linkage between changes in stream power and sediment supply inferred from the sedimentary record, finally, allowed us to detect possible shifts in concentrations of the supplied sediment during the sedimentation history of the Rigi megafan. It also yields information on the erosional processes and landscape forms in the hinterland where the sediment sources were situated (Fig. 3). Among the various parameters, the rate, the concentration and the granulometric composition of the supplied sediment represent first order independent variables that set the framework for establishing a sedimentary environment in sedimentary basins and that thus control the architecture of stratigraphic records. Our study illustrates that the extraction of the required information from fluvial conglomerates is possible and can be achieved through the quantification of first-order observations in the field, which includes the stacking pattern of conglomerates, channel depths, and data about grain size and clast petrography. As a further implication, our study reveals that causal links between erosional mechanisms operating on the surface, and geodynamic processes occurring at deep crustal levels can be established based on these first-order information preserved in the stratigraphic records.

## Methods

### Sedimentary environments

We identified the depositional mechanisms from the stacking pattern of conglomerate beds and the sedimentary structures (massive, cross-beds, imbrication, erosional scours) within the lithofacies^49^. Mudstone interbeds were described based on their sedimentary fabric (very fine lamination, ripple crossbeds, mottling, rootlets). In addition, we paid attention on noting the characterization of the surfaces separating conglomerates and mudstone interbeds (vertical scours, lateral fringing and gradual vertical transitions). Palaeo-flow directions were determined from clast imbricates and gutter casts where visible. Finally, the roundness of the clasts was qualitatively estimated using the template by Tucker^26^ as a basis.

We adapt the results of magneto-polarity^22^ and micromammal stratigraphies^50^ to constrain the chronological framework of the section. The resulting ages have an uncertainty of less than 0.5 Ma^22^.

### Estimation of palaeo-gradients of channels

**Figure 4:**
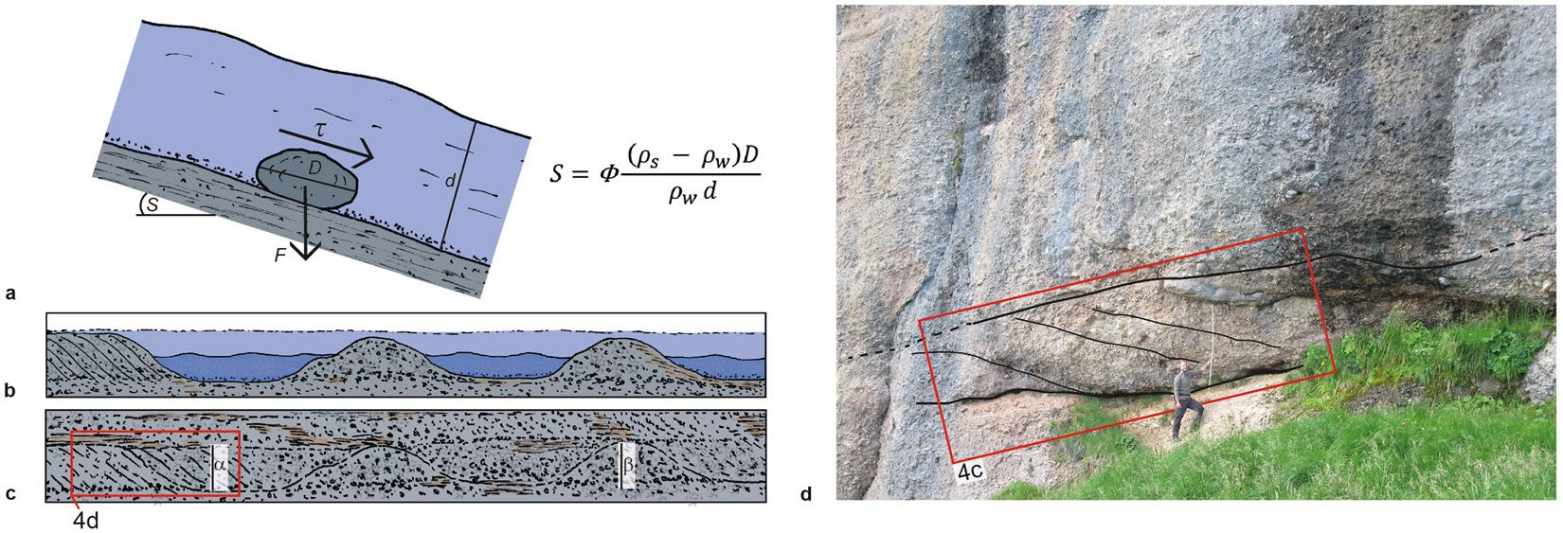
Balances of forces operating on a grain, and channel geometries. (**a**) Force balancing explaining the entrainment of sediment particles. A sediment particle with diameter *D* will be transported if the ratio between the fluid’s shear stress τ and the particle’s inertia force *F* ranges between 0.03 and 0.06, which is the Shields^27^ variable *ϕ*. These relationships can then be used to compute slope angles *S* at equilibrium conditions. The variable *d* denotes the water depth, *g* is gravitational acceleration, and *ρ*_*w*_ and *ρ*_*s*_ correspond to water and sediment densities, respectively. (**b**) Section through a braidplain illustrating water depths during low (dark blue) and bankfull discharge (pale blue), when all gravel bars bordering a channel are flooded. In this case, heights of foresets and longitudinal bars define the water depths during bankfull discharge conditions. (**c**) Illustration of how water depths representing bankfull discharge situations are registered in the sedimentologic archive. Accordingly, we measured the difference between the top of a conglomerate bank and the underlying erosional surface (α), and thicknesses of foresets and longitudinal bars (α, β). (**d**) Photo showing sediments of unit B. We used thicknesses of forests as proxy for channel depth, and we measured the largest clasts (*D*_*max*_) at the base of the foresets. Please see person and meter stick for scale.

Experimental^51^, numerical^5^ and geomorphic studies^4^ have disclosed positive correlations between energy gradients of streams, or alternatively river slopes *S*, sediment discharge *Q*_*s*_, sediment concentration *Q*_*c*_, and the grain size distribution of gravel bars. Here, we determined these variables and changes thereof using hydrological concepts for bedload entrainment. Related principles predict that a sediment particle with diameter *D* will be transported if the ratio between the fluid’s shear stress *τ* and the particle’s inertia force *F* ranges between 0.03 and 0.06 (Fig. 4a), which is referred to as the Shields^27^ variable *ϕ*. These *ϕ* values have been applied in numerous studies^8,52^ and consider a broad range of how clasts are arranged in bedload-dominated streams and how this affects the resistance to entrainment (e.g., loose and locked boundaries between clasts including hiding and protrusion effects^4,53,54^). Because our stream gradient calculations will heavily depend on hydrogeometric conditions at bankfull discharge (see below), we employed the range between 0.03 and 0.06 and a mean of 0.046 for the Shields variable *ϕ* ^55^,

Following Meyer-Peter and Müller^28^, the fluid’s shear stress depends on the hydrological radius, which corresponds to water depths *d* for wide channels, and the surface slope. As a consequence, the surface slope *S* can be computed using Shields criteria for the incipient motion of bedload particles paired with data about channel depths and grain size to be collected in the field^5^. Consequently, we used the aforementioned variables to solve eq. 27 by Meyer-Peter and Müller^28^ for sediment transport:

6 $$S=\varphi \frac{({\rho }_{s}-{\rho }_{w})D}{d{\rho }_{w}}$$

Here, the variables *ρ*_*s*_ and *ρ*_*w*_ denote the sediment and water densities, respectively. Slightly modified versions of this equation have been applied in theoretical^56^ and field-based studies^8^. However, a non-unique solution for equation (6) requires that for a given grain size, the corresponding water depth can be independently constrained by observations in the field^8^. Accordingly, we employ the sizes of the maximum clasts *D*_*max*_ paired with water depths during bankfull discharge *Q*_*b*_ to calculate the channel gradients *S*. The next paragraphs justify this approach and present details of how these variables were extracted from conglomerates.

### Estimates of water depth d from stratigraphic records

Among the various possibilities of water depth, the situation during bankfull discharge *Q*_*b*_ is considered to offer the most suitable conditions for solving eq. (6) for hydrological and sedimentologic reasons. First, it has been reported that *Q*_*b*_ of streams lays generally, but not always, in the same range as the mean annual floods of these rivers^39,56^, but we note that return periods of up to 2 years have also been reported^57^. This suggests that *Q*_*b*_ closely approximates the mean annual discharge conditions of a stream. In addition, water shear stresses of *Q*_*b*_ floods are large enough to mobilize nearly all grain size fractions of the bed material, resulting in equal mobility conditions^39^ including the largest clasts.

From a stratigraphic perspective, sedimentary archives representing *Q*_*b*_ conditions are best recorded and thus visible in the field through distinct arrangements of channels and gravel bars^58^, and have thus been used in comparable studies where the scope was to invert palaeo-slope conditions from stratigraphy^8^. Here, we used two proxies recorded by the Rigi conglomerates to measure water depths during bankfull discharge for the reach scale (Fig. 4b,c). These include the difference between the top of a conglomerate bank and the lowest point of juxtaposed and underlying erosional surfaces, and thicknesses of foresets (α) and longitudinal bars (β). While we cannot address the full range of palaeo-geomorphic complexities upon data collection (under- and overestimates of *d* related to uncertainties regarding e.g., main vs. tributary channel and post depositional erosion^56,58^), possible uncertainties related to our approach will not alter the general decreasing trend of flow depth from the base to the top of the Rigi section. Furthermore, upon measuring cross bed thicknesses, we paid special attention on selecting those units where topsets are preserved (Fig. 4d), thereby avoiding biases related to post depositional erosion^59^. In the same sense, conglomerate beds with a massive structure, which are indicative for longitudinal bars, are considered as proxy for defining bankfull channel depths only if basal erosional scours at the base and sand lenses on top of these bars are encountered. As sand is frequently deposited on top on longitudinal bars during waning floods, this approach ensures that underestimates related to post-depositional erosion are avoided^56,58^.

### Estimates of grain size D and related distributions from stratigraphic records

While most studies base their analyses on the *D*_*50*_ to solve the Meyer-Peter and Müller equation^28^ for palaeo-slopes^8,56^, we do not consider this as a valuable approach for the context of this paper, mainly because the Meyer-Peter and Müller equation^28^ bases on critical conditions for the entrainment of sediment. Indeed, because shear stresses during bankfull discharge floods *Q*_*b*_ are large enough to result in equal mobility conditions of grains across all percentiles including the *D*_*max*_ as field-based data has shown^39^, the selection of the *D*_*50*_ violates the criticality conditions that are included in Shields’ criteria. We thus employ the *D*_*max*_ to solve equation (6) also because a one-to-one correlation between fan surface slopes and *D*_*max*_ have been reported from modern fans at the foothill of the Tucson Mountains and the Aubrey Cliff, AZ^60^. We proceeded by measuring the size of the 4 to 6 largest pebbles and boulders^53^ at the tip of those bars where we took palaeo-channel depths measurements (Fig. 4d). We then used the mean of these values to infer the size of the *D*_*max*_ at these sites.

Ideally, the Meyer-Peter and Müller solution^28^ for sediment transport applies to grains for which the *b-* (intermediate) axis has been determined, which decently approximates the nominal diameter of ellipsoidal clasts^61^. However, 2D-exposures of conglomerate outcrops preclude a full 3D view on the clasts in the sense that they either expose the *a-b* or *b-c* pairs of axes only. Therefore, unambiguous identifications of *b-*axes are not possible. Among the various possibilities, *b/a* ratios of c. 0.7 have been measured on >3000 clasts in streams surrounding the Central European Alps^38^, while no quantitative data is available on neither of the ratios involving the *c*-axis. Therefore, we avoided data collection in the field that involved measurements of the *c-*axes because their relationships to the *b-*axes remain unknown and are thus associated with large and non-quantifiable uncertainties. This leaves us with a strategy, where we measured the longest axis in field. We then accounted for biases related to the exposure conditions through a 15% reduction of the measured lengths, thereby considering the *b/a* ratios of 0.7^38^ and the 50% chance of exposure of either axis.

Here, we justify the aforementioned correction through the following thought experiment: Provided that we did indeed measure the *b-*axis (50% chance), our correction yields in a 15% underestimate of the real length of the *b-*axis. In case we measured the *a*-axis (again 50% chance), then our approach overestimates the length of the *b-*axis by 15%. As the mean of 4 to 6 largest clasts were determined per site, this strategy returns a value that closely corresponds to the b-axis to be considered for the calculation of the slopes (eq. 6). However, since the discussion of this paper mainly focuses on vertical shifts in stratigraphic records and because uncertainties in the *D*_*max*_ linearly propagate through our analyses according to eq. (6), we do not consider that a related bias will alter the here presented scenarios and interpretations.

We determined the sediment calibre for c. 50 sites, where we measured a total of approximately 4500 grains using the Wolman^62^ clast counting method. Here, we were faced with the same problem as in the field because digital images do not allow to unequivocally assign a-, b- or c-axes of clasts. However, since we are mainly interested in the vertical grain size trends, we do not consider that this issue will bias related interpretations.

### Data Availability

The datasets generated during the current study are available for free from the corresponding author on request.
